# Interleukin-1 Induces the Release of Lubricating Phospholipids from Human Osteoarthritic Fibroblast-Like Synoviocytes

**DOI:** 10.3390/ijms23052409

**Published:** 2022-02-22

**Authors:** Vishnu Thottakkattumana Parameswaran, Christiane Hild, Gerrit Eichner, Bernd Ishaque, Markus Rickert, Juergen Steinmeyer

**Affiliations:** 1 Laboratory for Experimental Orthopaedics, Department of Orthopaedics, Justus Liebig University Giessen, 35392 Giessen, Germany; vishnu.thottakkattumana-parameswaran@ortho.med.uni-giessen.de (V.T.P.); christiane.hild@ortho.med.uni-giessen.de (C.H.); bernd.ishaque@ortho.med.uni-giessen.de (B.I.); markus.rickert@ortho.med.uni-giessen.de (M.R.); 2 Mathematical Institute, Justus Liebig University Giessen, 35392 Giessen, Germany; gerrit.eichner@math.uni-giessen.de

**Keywords:** phospholipids, interleukin, LXR, ABCA1, cholesterol hydroxylase, CH25H, CYP7B1, FLS, synovial fibroblasts, osteoarthritis

## Abstract

(1) Background: Synovial fluid (SF) from knee joints with osteoarthritis (OA) has increased levels of phospholipids (PL). We have reported earlier that TGF-ß and IGF-1 stimulate fibroblast-like synoviocytes (FLS) to synthesize increased amounts of PLs. The current study examined whether IL-1ß induces the release of PLs in FLS and the underlying mechanism. (2) Methods: Cultured human OA FLS were treated with IL-1ß alone and with pathway inhibitors or with synthetic liver X receptor (LXR) agonists. Cholesterol hydroxylases, ABC transporters, apolipoproteins (APO), LXR, sterol regulatory binding proteins (SREBPs), and 3-hydroxy-3-methylglutaryl-coenzyme A reductase (HMGCR) were analyzed by RT-PCR, Western blot, and ELISA. The release of radiolabeled PLs from FLS was determined, and statistical analysis was performed using R (N = 5–9). (3) Results: Like synthetic LXR agonists, IL-1ß induced a 1.4-fold greater release of PLs from FLS. Simultaneously, IL-1ß upregulated the level of the PL transporter ABCA1 and of cholesterol hydroxylases CH25H and CYP7B1. IL-1ß and T0901317 stimulated the expression of SREBP1c, whereas only T0901317 enhanced SREBP2, HMGCR, APOE, LXRα, and ABCG1 additionally. (4) Conclusions: IL-1ß partially controls PL levels in OA-SF by affecting the release of PLs from FLS. Our data show that IL-1ß upregulates cholesterol hydroxylases and thus the formation of oxysterols, which, as natural agonists of LXR, increase the level of active ABCA1, in turn enhancing the release of PLs.

## 1. Introduction

The nearly frictionless motion of joints is enabled by a thin layer of complexed macromolecules that comprise phospholipids (PLs), hyaluronan, and lubricin, which coat the articulating surfaces and are replenished by synovial fluid (SF) [1]. We have reported that in osteoarthritis (OA) and rheumatoid arthritis, the levels of PLs in SF increase significantly [2,3]. However, hemifusion of the exposed lipid bilayers [4] and the simultaneous decrease in lubricin and hyaluronan, as observed in SF during OA [5], can result in higher friction at the cartilage boundary layers.

PLs in SF are derived in part from synovial blood vessels and can also be produced and possibly secreted by fibroblast-like synoviocytes (FLS). Using cultured human OA FLS in a previous study, we showed that transforming growth factor-ß1 (TGF-ß1) and insulin-like growth factor-1 (IGF-1) stimulate the biosynthesis of the major PL phosphatidylcholine (PC), whereas interleukin-1ß (IL-1ß) enhances that of phosphatidylethanolamine and plasmalogens [6,7].

A total of 20 of 48 human ATP-binding cassette (ABC) transporters are involved in the transport of lipids and lipid-like molecules and are in turn regulated by PLs, fatty acids, and sterols [8,9]. ABCA1 is crucial for the release of cellular cholesterol and PLs to apolipoprotein (APO) A1 (APOA1)-containing high-density-lipoprotein (HDL)-containing precursors, which constitutes the first step in reverse cholesterol transport (RCT). In addition, cellular ABCG1 has a significant function in the transfer of cholesterol to HDLs. According to a 2-step model, ABCA1 promotes the release of cholesterol and PLs that are involved in membrane microdomains to lipid-poor APOA1. Subsequently, the preformed PL-protein complex is loaded with additional cellular cholesterol by ABCG1 [9,10].

The release of lipids is controlled through the tight regulation of the transcription and translation of transporters [10]. The 48 nuclear receptors constitute a family of ligand-activated transcription factors that, on binding to specific sites in DNA, recruit transcriptional machinery to influence gene expression. The nuclear oxysterol receptors liver X receptor (LXR) and retinoid X receptor (RXR) form heterodimers on stimulation that also bind a promoter sequence to initiate the transcription of the ABCA1, ABCG1, and APOE genes to downregulate cholesterol overload [11,12,13,14]. LXR functions primarily as a cholesterol sensor that is activated by endogenous oxysterols that form proportionally with the cellular cholesterol content [11]. LXRα and LXRß, encoded by nuclear receptor subfamily 1 group H members 2 and 3 (NR1H2 and NR1H3, respectively), differ in tissue distribution but are expressed in joints [11,12,15].

Human OA chondrocytes contain lipid droplets and express lower levels of LXRs, ABCA1, and APOA1 involved in cholesterol efflux [12,15]. The synthetic LXR agonist T0901317 reverses this impairment in cholesterol release by upregulating ABCA1 and APOA1 [15]. LXRs also regulate catabolic proteases, inflammation, and immunity [16,17]. In response to a synthetic LXR agonist, the expression of MMP13 and ADAMTS4 in chondrocytes and of IL-6, COX-2, and iNOS in macrophages declines [12,16,17]. Further, oral treatment with LXR agonists improves arthritis symptoms and inhibits pain and extracellular matrix degradation in animal models of OA [18,19]. Thus, nuclear LXR has garnered significant attention as a pharmacological target for OA and other inflammatory diseases [11,14,20]. Four synthetic LXR agonists, including T0901317 and GW3965, advanced to phase I clinical trials but were not developed further due to side effects, such as hepatic steatosis and hypertriglyceridemia [11,21].

Oxysterols, such as the oxygenated cholesterol derivate 25-hydroxycholesterol (25-HC), are endogenous agonists of LXRs. 25-HC is generated by the endoplasmic reticulum enzyme cholesterol 25-hydroxylase (CH25H). 25-HC can be hydroxylated further to 7α,25-dihydroxycholesterol (7α,25-HC) by the monooxygenase 25-hydroxycholesterol 7-alpha-hydroxylase also called cytochrome P450 7B1, which is encoded by CYP7B1. Recently, Choi et al. [22] reported that OA chondrocytes contain elevated levels of cholesterol due to augmented uptake and upregulation of CH25H and CYP7B1, increasing the content of oxysterols [23]. They also showed that adenoviral overexpression of either hydroxylase causes OA in mice and that both hydroxylases are upregulated in human OA cartilage [22]. In addition, the proinflammatory cytokines IL-1ß and TNFα induce the mRNA expression of both cholesterol hydroxylases and, subsequently, 25-HC and 7α,25-HC in murine chondrocytes [22].

In addition to blood vessels, articular cells, such as FLS, are considered a possible source of PLs in SF. However, it remains unknown whether and how FLS releases PLs and thus contributes to the elevation of PLs in OA-SF. In this in vitro study, we hypothesized that IL-1ß stimulates the release of PLs from FLS, mediated by the upregulation of cholesterol hydroxylases and ABC transporters. We found that the binding of agonists to LXR and the post-translational effects of IL-1ß enhance the expression of ABCA1 transporters, resulting in greater release of PLs from FLS.

## 2. Results

### 2.1. IL-1ß and LXRα Agonists Induce Greater Release of PLs from FLS

**Figure 1:**
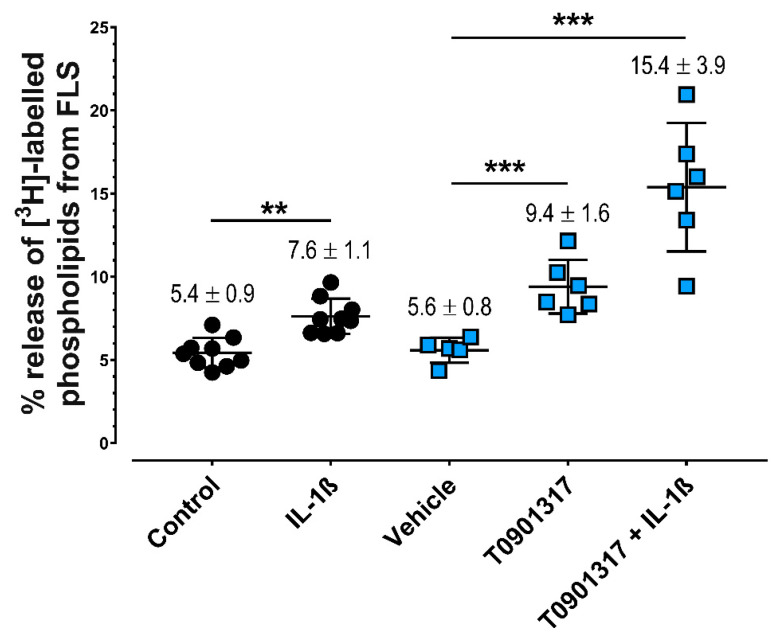
IL-1ß and a synthetic LXR agonist induce the release of radiolabeled PLs from FLS. Release of [^3^H]-labeled PLs into the media is expressed as percentage of PLs in media from total PLs (media + cell lysate) after normalization to total cellular protein content. FLS were treated for 48 h with 1.0 ng/mL IL-1ß or 0.1 µM T0901317 ± 1.0 ng/mL IL-1ß. Data for biological replicates are shown as dot plots, with lines inside showing the mean ± SD (*n* = 5–9). Black circles represent data from IL-1ß-treated FLS versus control, whereas blue squares represent data from T0901317 ± IL-1ß versus vehicle control. ** *p* < 0.01, *** *p* < 0.001.

Treatment of FLS with IL-1ß increased the release of radiolabeled PLs into the media by 1.40 ± 0.33-fold (*p* = 0.007) (Figure 1). This induction was not significantly affected by the addition of the NF-κB inhibitor QNZ (7.8% ± 1.1%, *p =* 0.99), the p38 MAPK inhibitor SB203580 (8.3% ± 1.8%, *p* = 0.98), or the broad-spectrum JNK inhibitor SP600125 (8.3% ± 2.9%, *p* = 0.93). However, 0.1 µM T0901317 stimulated the release of labeled PLs by 1.56 ± 0.17-fold (*p* ≤ 0.001), which was enhanced further by 1.0 ng/mL IL-1ß (Figure 1). Administration of another synthetic LXR agonist, GW3965, confirmed this stimulatory effect, this time to the tune of 1.78 + 0.25-fold compared with untreated controls (GW3965: 10.0% ± 1.2%; vehicle: 5.7% ± 0.9%, *p* < 0.001). Similarly, this increase in PL release was further enhanced by 1.0 ng/mL IL1ß to 2.96 + 0.66-fold (GW3965: 16.5% ± 2.1%, *p* < 0.001). 

### 2.2. Cholesterol Hydroxylases Are Upregulated by IL-1ß

**Figure 2:**
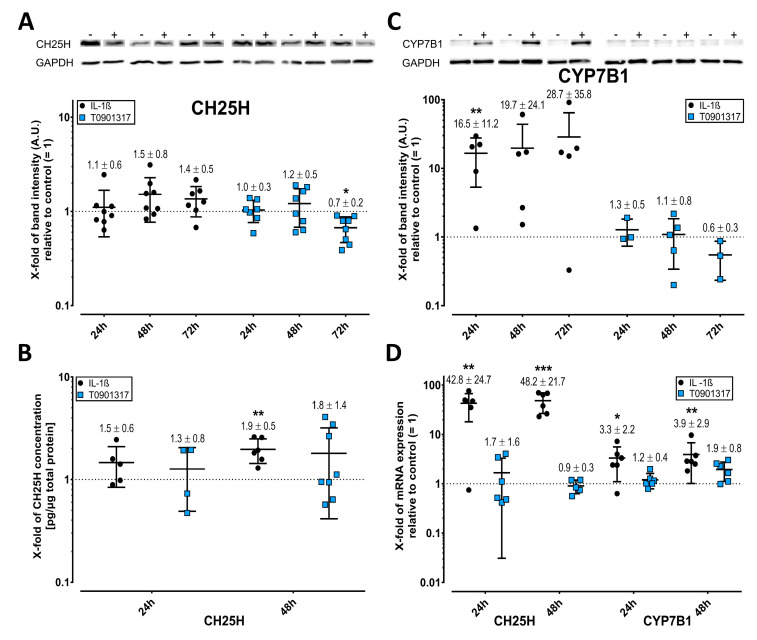
IL-1ß induces the expression of cholesterol hydroxylase CH25H and CYP7B1 in FLS. CH25H (**A**) and CYP7B1 (**C**) were measured by Western blot or enzyme-linked immunosorbent assay (**B**). The mRNA expression of CH25H and CYP7B1 (**D**) was determined by reverse transcription-polymerase chain reaction (RT-PCR) using the 2^−ΔΔCt^ method. Representative western blots show the protein expression of cultured FLS as the corresponding control (−) and of FLS treated (+) with IL-1ß or T0901317. All western blots were quantified using ImageJ (**A**,**C**). Treated FLS and corresponding controls derived from the same experiment, and patient and gels/blots were processed in parallel. FLS were treated with 1 ng/mL IL-1ß or 0.1 µM T0901317 for 24, 48, and 72 h. Data for biological replicates represent fold-change versus corresponding controls (= 1, indicated by broken horizontal lines) and are shown as dot plots, with lines inside showing the mean ± SD (*n* = 8). The y-axis represents the log of variable fold changes. Black circles show data from IL-1ß-treated FLS relative to untreated controls, whereas blue squares represent data from T0901317 relative to vehicle controls. * *p* < 0.05, ** *p* < 0.01, *** *p* < 0.001.

Oxysterols, such as 25-HC and 7α,25-HC, are oxidized cholesterol derivatives that are generated by CH25H and CYP7B1, respectively. By Western blot, IL-1ß induced CH25H and CYP7B1 (Figure 2A,C), and ELISA confirmed the IL-1ß induced CH25H expression after 48 h (Figure 2B). After 24 and 48 h, IL-1ß upregulated the mRNA levels of CH25H and, to a lesser extent, CYP7B1 (Figure 2D). Relative to IL-1ß alone, the addition of QNZ or SP600125 did not significantly reverse the effects of IL-1ß on CH25H mRNA (FDR-adjusted *p =* 0.41). However, QNZ significantly upmodulated CYP7B1 mRNA (*p* < 0.001) in the presence of IL-1ß, which appears to be a substance-specific effect. The synthetic LXR agonist T0901317 did not affect the mRNA levels of either cholesterol hydroxylase (Figure 2D).

### 2.3. LXR Expression Is Enhanced by T0901317 but Not IL-1ß

**Figure 3:**
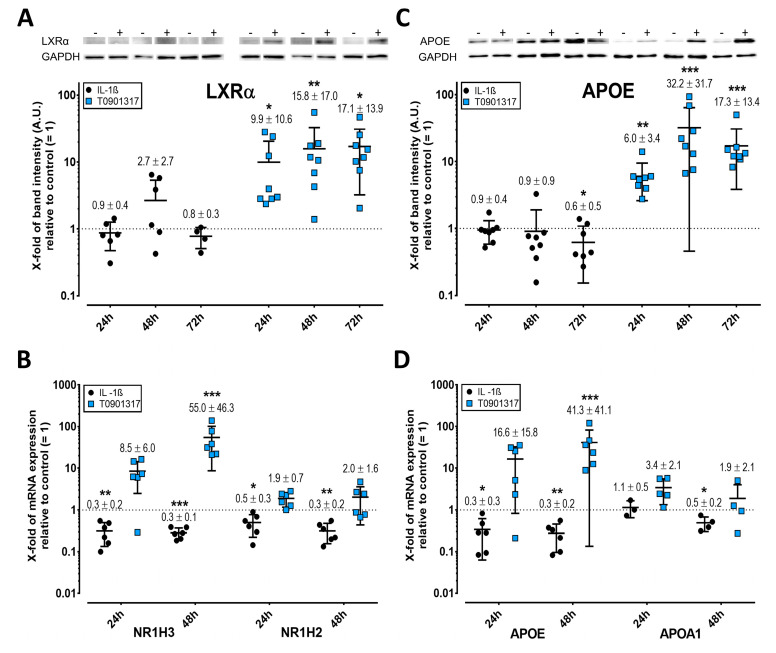
T0901317 but not IL-1ß induces the expression of liver X receptor (LXR) and apolipoprotein E (APOE) in FLS. Western blots of LXRα (**A**) and APOE (**C**). The relative mRNA expression of LXRα and LXRß (**B**) and APOA1 and APOE (**D**) was determined by RT-PCR using the 2^−ΔΔCt^ method. For further details, see the legend for Figure 2, which is fully analogous, but here for LXRα and ß, APOA1, and APOE. * *p* < 0.05, ** *p* < 0.01, *** *p* < 0.001.

Oxysterols are endogenous agonists of LXR. Western blots showed that LXRα expression remained unchanged upon treatment with IL-1ß, whereas, however, the strong stimulatory effect of T0901317 on mRNA expression was confirmed (Figure 3A). Treatment of FLS with IL-1ß for 24 and 48 h significantly inhibited the mRNA expression of both LXR isoforms, whereas T0901317 upregulated LXRα, but not LXRß (Figure 3B). The addition of QNZ or SP600125 did not significantly mitigate the effects of IL-1ß alone on NR1H3 (FDR-adjusted *p* = 0.50) or NR1H2 mRNA (FDR-adjusted *p* = 0.50).

### 2.4. APOE but Not APOA1 Is Upregulated by T0901317

APOE mRNA and protein were significantly stimulated by T0901317, whereas IL-1ß inhibited them (the latter after 72 h) (Figure 3C,D). APOA1 mRNA was downregulated after 48 h with IL-1ß, whereas T0901317 had no effect (Figure 3D). QNZ and SP600125 did not significantly alter the mRNA levels of APOA1 (FDR-adjusted *p* = 0.99) or APOE (FDR-adjusted *p* = 0.76) versus IL-1ß alone.

### 2.5. Elevated ABC Transporter Expression after Treatment with IL-1ß and T0901317

**Figure 4:**
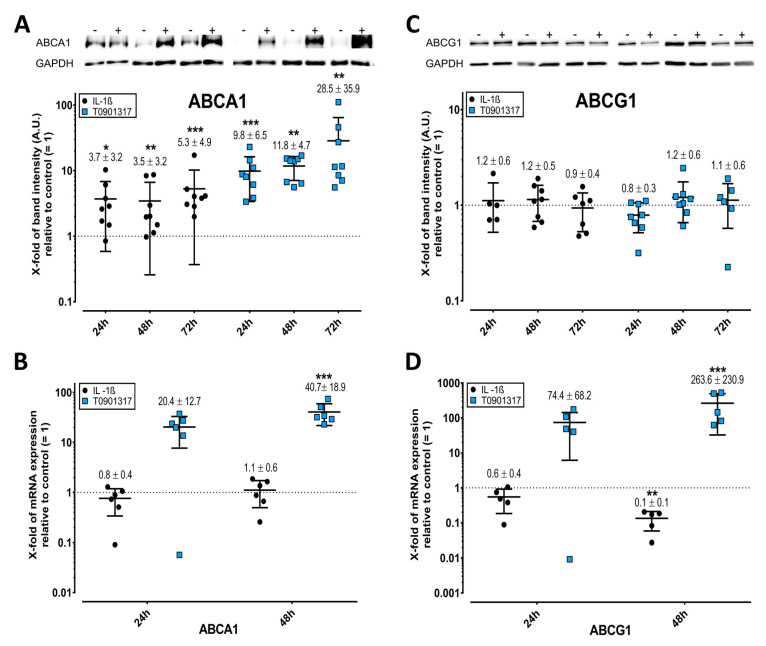
IL-1ß and T0901317 induce the expression of ABCA1 but not ABCG1 in FLS. Western blots of ABCA1 (**A**) and ABCG1 (**C**). The mRNA expression of ABCA1 (**B**) and ABCG1 (**D**) was determined by RT-PCR using the 2^−ΔΔCt^ method. For further details, see the legend for Figure 2, which is fully analogous, but here for ABCA1 and ABCG1. * *p* < 0.05, ** *p* < 0.01, *** *p* < 0.001.

The cholesterol and PL transporters ABCA1 and ABCG1 are central to the release of active PLs. IL-1ß upregulated ABCA1 protein but not mRNA, whereas T0901317 stimulated both (Figure 4A,B). However, neither compound altered the protein expression of ABCG1 (Figure 4C), despite IL-1ß inhibiting and T0901317 upregulating its mRNA. QNZ and SP600125 did not significantly mitigate the effects of IL-1ß alone on ABCG1 mRNA (FDR-adjusted *p* = 0.76).

### 2.6. Opposing Effects of T0901317 and IL-1ß on SREBPs and HMGCR

**Figure 5:**
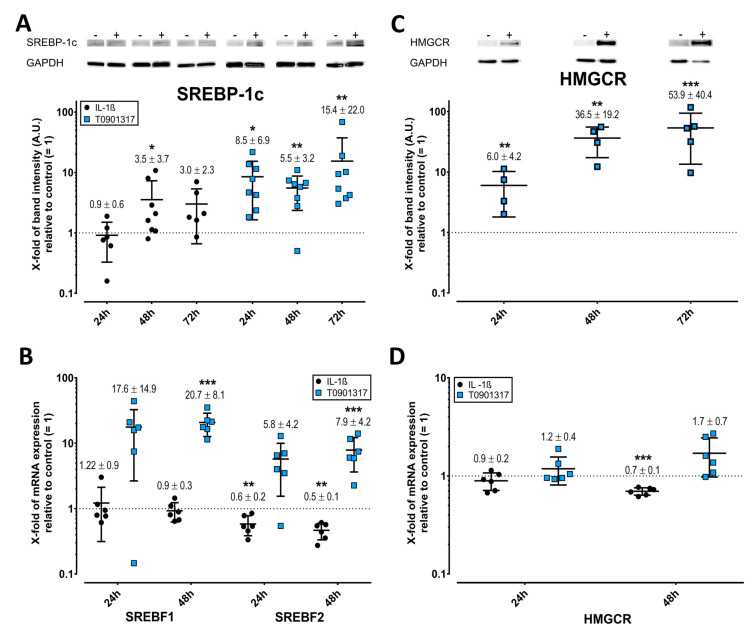
Effect of IL-1ß and T0901317 on the expression of 3-hydroxy-3-methylglutaryl-CoA reductase (HMGCR) and sterol regulatory element-binding protein (SREBP) 1c and 2 in FLS. Western blots of SREBP1c (**A**) and HMGCR (**C**). The mRNA expression of SREBF1 and 2 (**B**) and HMGCR (**D**) was determined by RT-PCR using the 2^−ΔΔCt^ method. For further details, see the legend for Figure 2, which is fully analogous, but here for both SREBPs/SREBFs and HMGCR. * *p* < 0.05, ** *p* < 0.01, *** *p* < 0.001.

T0901317 significantly upregulated SREBP-1C, SREBP2, and HMGCR, which is the rate-controlling enzyme of the mevalonate pathway (Figure 5A–C). Notably, SREBP-1c expression was slightly elevated on treatment with IL-1ß by Western blot (Figure 5A). However, IL-1ß significantly inhibited the transcription of SREBF2 and HMGCR, compared with untreated control (Figure 5B,D), rendering them undetectable by Western blot. Relative to the sole effects of IL-1ß, the addition of pathway inhibitors either significantly inversed the mRNA expression (HMCR, *p* < 0.01) or not (SREBF2, FDR-adjusted *p* = 0.35).

## 3. Discussion

**Figure 6:**
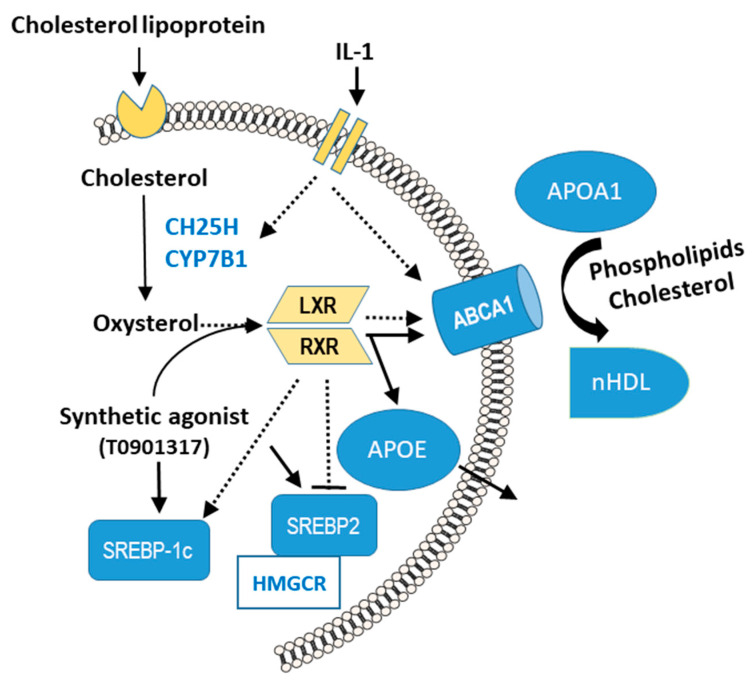
Schematic of proposed mechanism of IL-1ß on phospholipid (PL) release from FLS during OA. IL-1ß induces the upregulation of cholesterol 25-hydroxylase (CH25H) and monooxygenase 25-hydroxycholesterol 7-alpha-hydroxylase, also called cytochrome P450 7B1 (CYP7B1), causing accumulation of oxysterols as endogenous ligands of nuclear liver X receptor (LXR). On activation of LXR by oxysterols or synthetic LXR agonists, such as T0901317, the protein level of active ATP-binding cassette transporter A1 (ABCA1) increases. The increased efflux of PLs to extracellular apolipoprotein (APO) A1 is mediated by ABCA1, which results in the formation of nascent HDL-c particles (nHDL). Oxysterols and T0901317 upregulate sterol regulatory element-binding protein (SREBP) 1c, suggesting that the biosynthesis of fatty acids that are needed for PLs is stimulated. Further, T0901317 enhances the expression of SREBP2, which binds as an important transcription factor to the promoter region of 3-hydroxy-3-methylglutaryl-coA reductase (HMGCR), the rate-limiting enzyme in cholesterol biosynthesis, to upregulate it. Dotted line: IL-1; solid line: T0901317; arrow: stimulation; dash: inhibition.

FLS supplies SF with lubricating compounds, such as lubricin and hyaluronan [5,24]. SF in OA contains elevated levels of lubricating PLs [2,3], and TGF-ß1 and IGF-1 stimulate FLS to synthesize increased amounts of the major PL class PC [7]. In this study, we found that IL-1ß stimulates FLS to release significantly higher levels of PLs. One of our central findings is that IL-1ß induces the release of PLs by upregulating cholesterol hydroxylase CH25H and CYP7B1, resulting in the formation of natural oxysterols, which are LXR agonists such as the synthetic T0901317. The oxysterols and T0901317 significantly increased the level of active ABCA1, which subsequently transported higher amounts of PLs to APOA1 (Figure 6).

A widely held view is that IL-1ß contributes to the pathogenesis of OA in an autocrine and paracrine manner, particularly during inflammatory phases [25,26]. This cytokine is produced in articular joints by various cells such as chondrocytes, monocytes, and macrophages of synovium, adipocytes, and osteoblasts [27]. In addition, elevated levels of IL-1ß have been detected in SF, synovial membrane, subchondral bone, and cartilage in OA patients [25,28]. Choi et al. [22] demonstrated that IL-1ß stimulates mouse chondrocytes and FLS to transcribe CH25H and CYP7B1, increasing the levels of the corresponding oxysterols, 25-HC and 7α,25-HC, which are natural LXR agonists. LXRα and LXRß mRNA, alone and in tandem, is downregulated in OA versus normal cartilage, whereas IL-1ß has stimulatory and inhibitory effects [12,15,22]. In contrast, our study shows that IL-1ß also inhibits the mRNA of both LXR isoforms but not LXRα protein expression. However, none of the specific inhibitors of the P38 MAPK, NF-κB, or JNK pathway could antagonize IL-1ß-induced PL release or CH25H expression, necessitating a more in-depth study of the underlying signaling. Further, the pathway inhibitors had no effect on IL-1-altered mRNA expression of LXR, apolipoproteins, or ABCG1, suggesting an oxysterol-mediated effect of IL-1ß.

Notably, IL-1ß treatment did not alter ABCA1 mRNA levels but upregulated its protein. This disparity indicates that in FLS, the level of ABCA1 protein is controlled by IL-1ß through post-translational mechanisms. In vitro studies with modest elevated cellular cholesterol, for example, have shown that ABCA1 levels are regulated by the rate of degradation [29,30]. Further, LXRß has a nongenomic function, wherein a fraction of cytosolic LXRß binds to the C-terminal region of plasma membrane-bound ABCA1, forming a stable but inert ABCA1-LXRß/RXR complex and thus retarding ABCA1 degradation [31,32]. Once cholesterol accumulates, the production of oxysterols, such as 25-HC and 7α,25-HC, rises. Oxysterols bind to LXRß as agonists, causing the complex to dissociate from ABCA1, rendering ABCA1 functional and able to bind to APOA1 and thus leading to subsequent cholesterol and PL efflux [10,31,32]. Collectively, our data indicate that IL-1ß post-translationally elevates the level of active non-complexed ABCA1, enhancing the release of PLs.

In contrast to the synthetic LXR agonist T0901317, the IL-1ß-induced 25-HC, respectively, 7α,25-HC did not significantly upregulate the LXR target genes APOE, ABCG1, and ABCA1 (Figure 6). The oxysterol 25-HC is a weak LXR agonist [33] that, with its slightly elevated expression, might have effected insufficient activation and binding of LXR to LXR-responsive elements in the promoters of ABCA1, ABCG1, and APOE. However, our data on T0901317 are consistent with reports that synthetic LXR agonists enhance the biosynthesis of APOE in human OA chondrocytes and macrophages and in adipose tissue [12,34,35]. We assume that APOE is secreted by FLS, in which it is involved as a constituent of plasma lipoproteins in the transport and metabolism of PLs, cholesterol, and triacylglycerols [36]. Because cholesterol autoxidizes in vitro and due to the difficulty of experimenting with oxysterols at physiological levels, we used two synthetic agonists of LXR (T0901317 and the more specific GW3965) for comparison.

SREBP2 preferentially activates such genes as HMGCR, the key enzyme of cholesterol biosynthesis. Oxysterols fine-tune cellular cholesterol homeostasis by controlling the release of cholesterol via LXR activation and cholesterol synthesis through blockade of SREBP2 activation, downregulating HMGCR [11,35,37]. Activation of LXRs by oxysterols upregulates SREBP1c and subsequently the transcription of genes that mediate fatty acid synthesis, which is needed for the biosynthesis of PLs [38]. These results are consistent with our data, wherein IL-1ß stimulated the biosynthesis of the LXR target gene SREBP1c but inhibited SREBP2 and HMGCR, likely through oxysterols (Figure 6). Our data imply that IL-1ß contributes to the increased articular production of PLs during OA through stimulated fatty acid synthesis, as reported for OA FLS [6,7].

However, T0901317 significantly upregulated SREBP1c, SREBP2, and HMGCR, indicating a compound-specific effect, in which this LXR agonist uncoupled the control of cholesterol release from biosynthesis. Our data are consistent with earlier reports that the systemic application of synthetic LXR agonists induces lipogenic genes in the liver, leading to hepatic steatosis and hypertriglyceridemia [11].

There are several limitations of our study. Screening for a concentration-dependent effect of IL-1ß would have strengthened our results. However, using FLS from nine patients, the IL-1ß-induced enhanced release of PLs was reproducibly measured. In addition, the marked upregulation of ABCA1 transporter protein in IL-1ß-treated FLS might explain the greater release in PLs. Further, the mRNA and protein levels of several parameters were discordant. However, the use of mRNA patterns alone is insufficient to understand protein expression because posttranscriptional mechanisms, including protein translation, post-translational modifications, and degradation, can affect the concentration of proteins in cells and tissues. Thus, we focused our discussion on the data on the expression of proteins.

In conclusion, we have demonstrated novel results that the proinflammatory cytokine IL-1ß enhances PL release from FLS by elevating the levels of the active noncomplexed transporter ABCA1. Our data imply that IL-1ß upregulates the cholesterol hydroxylases CH25H and CYP7B1 and the generation of the oxysterols 25-HC, respectively, 7α,25-HC. We assume that LXRß, on binding with their natural agonist oxysterol, dissociates from ABCA1, enabling the release of PLs and cholesterol. Collectively, our current and previous results suggest that during OA, growth factors, such as TGF-ß1 and IGF1, trigger the generation of PLs, whereas IL-1ß controls their release from FLS. These findings might constitute an attempt of OA articular joints to compensate for the pathologically low levels of the lubricants hyaluronan and lubricin in SF.

## 4. Materials and Methods

### 4.1. Source of Fibroblast-Lile Synoviocytes

FLS were derived from the synovial tissue of OA patients who were undergoing total knee arthroplasty in the Department of Orthopaedics of the University Hospital Giessen (Germany). Approval by the ethical review committee (Faculty of Medicine, Justus Liebig University Giessen, Germany) was obtained, and written informed consent was provided by donors. The exclusion criteria were other joint diseases, such as rheumatoid arthritis, gout, and trauma; HIV infection, tumors near the joint; severe liver and kidney disease; drug abuse; and a history of treatment with immunosuppressive drugs, corticosteroids, or hyaluronan in the preceding 6 months.

### 4.2. Isolation, Cell Propagation, and Purity of FLS

FLS were isolated from synovial membranes as described [39]. FLS were propagated until Passage 4 in a humidified atmosphere at 37 °C with 10% CO_2_ using Dulbecco’s Modified Eagle’s Medium (DMEM) that was supplemented with 10% fetal bovine serum (FBS), 10 mM HEPES buffer, 100 U/mL penicillin, and 0.1 mg/mL streptomycin, 1.0 g/L glucose, and 584 mg/mL L-glutamine.

The purity of FLS was determined prior to treatment on a FACSCanto II (BD Biosciences, Heidelberg, Germany), confirming the presence of at least 90% (96.9 ± 3.2) of cells that stained positively for the fibroblast antigen CD90 using APC anti-human CD90 (clone 5E10, RRID: AB_893440, BioLegend, London, UK). Hematopoietic cells were negative against PE anti-human CD45 (clone 2D1, RRID: AB_2566369, BioLegend). The media were consistently tested negative for contamination with mycoplasma using the PCR Mycoplasma Test Kit I/C (PromoCell, Heidelberg, Germany).

### 4.3. Treatment of FLS to Study PL Release

To study the release of choline-containing PLs from FLS during treatment, confluent cells from Passage 5 were cultured in 6-well plates and labeled with 5 µCi/mL [^3^H]-choline chloride (PerkinElmer, Rodgau, Germany) for 24 h in choline-depleted, phenol red-free DMEM (PAN Biotech) that was supplemented as above. FLS were washed thoroughly to remove unincorporated precursors and starved for 24 h with supplemented DMEM that contained 5% FBS. After a change in media, cells were treated for 48 h with 0.1 µM T0901317 (Sigma-Aldrich, Taufkirchen, Germany) or GW3965 (Cayman Chemical Company, Ann Arbor, MI, USA); 1 ng/mL IL-1ß (PeproTech, Hamburg, Germany), and 1 µM of the P38 MAPK inhibitor SB203580, 0.1 µM of the NF-κB inhibitor QNZ, the broad-spectrum JNK inhibitor SP600125 (Selleck Chemicals, Munich, Germany), or a negative control. The media and treatment were changed after 24 h. The release of radiolabeled PLs was measured in media from the final 24 h of the experiment. The experiment was repeated 4–8 times using FLS from 5 to 9 OA patients (age 53–81 years (69.3 ± 8.2), BMI 26.8–34.9 kg/m^2^ (30.1 ± 2.4), Kellgren-Lawrence scores of 2–4 (3.1 ± 0.8), CRP 3.18 ± 4.51 mg/L, 3 males, 6 females).

### 4.4. Determination of Radiolabeled PLs

After treatment, FLS and media were collected separately. Cells were washed twice with 1x PBS and lysed in situ using 0.1% sodium dodecyl sulfate (SDS). Total lipids were extracted from 2 mL media and 400 µL of cell lysate by Bligh and Dyer method [40]. The chloroform phase that contained the radiolabeled PLs was mixed with liquid scintillation cocktail (Emulsifier-Safe™; PerkinElmer), and radioactivity was measured in triplicate on a multipurpose scintillation counter (LS 6500, Beckman Coulter, Krefeld, Germany). The quantitative dpm values were normalized to the cellular protein content, which was quantified with the Pierce™ BCA Protein Assay Kit (Thermo Fisher Scientific, Dreieich, Germany). The release of [^3^H]-labeled PLs from FLS into media was calculated as the percentage of radiolabeled PLs in the media relative to the total [^3^H]-labeled PLs in the media and cell lysate.

### 4.5. Treatment of FLS to Study PL Transport Mechanism

FLS from Passage 5 were cultured in 6-well plates until confluence and then starved in DMEM that was supplemented with 5% FBS for 48 h; the media was changed once after 24 h. Cells were treated with 0.1 µM T0901317 or 1 ng/mL IL-1ß with or without pathway inhibitors. To examine cellular PL transport, FLS were treated for 24 and 48 h for mRNA expression and an additional 72 h for protein expression, lysed in 0.1% SDS, and stored at −80 °C until analysis. The experiment was repeated 7 times using FLS from 8 OA patients (age 66–85 years (75.8 ± 5.96), BMI 22.8–30 kg/m^2^ (28.4 ± 2.71), Kellgren–Lawrence scores of 2–4 (3.0 ± 0.5), CRP 3.95 ± 4.95 mg/L, 3 males, 5 females).

### 4.6. Analysis of mRNA Expression

Total RNA from FLS was extracted using peqGOLD TriFast (VWR International) according to the manufacturer’s instructions. The amount and purity of total RNA were analyzed on a NanoDrop™ 2000 (Thermo Fisher), and the RNA samples in our RT-PCR analysis had a 260/280 nm ratio of 1.96 ± 0.03. Total RNA was reverse-transcribed using the QuantiNova Reverse Transcription Kit (Qiagen, Hilden, Germany) as per the manufacturer’s instructions. Expression studies were performed with the cDNA obtained and the QuantiNova SYBR Green PCR kit (Qiagen) using the 7500 Fast Real-Time-PCR system (Applied Biosystems™, Thermo Fisher Scientific, Dreieich, Germany).

The following QuantiTect Primer assays (Qiagen) were used to measure the mRNA expression according to the manufacturer’s instructions: CH25H (QT00202370), CYP7B1 (QT00079485), NR1H2 (QT00057967), NR1H3 (QT00065156), ABCA1 (QT00064869), ABCG1 (QT00021035), APOA1 (QT00015841), APOE (QT00087297), sterol regulatory element-binding transcription factor (SREBF) 2 (SREBF2, QT00052052), SREBF1 (QT00036897), and 3-hydroxy-3-methyl-glutaryl-coenzyme A reductase (HMGCR, QT00004081). QuantiTect Primer assays are predesigned primer pairs that are bioinformatically validated and highly specific for the target cDNA.

The efficiency of amplification of all primer pairs was 97% to 113% (104.3 ± 4.7) obtained by the serial dilution method [41]. The relative expression of each target gene was calculated versus GAPDH (QT01192646) and an untreated control by the 2^−ΔΔCt^ method.

### 4.7. ELISA of CH25H

FLS lysates (20 µg of proteins) were obtained after 48 h and concentrated 3 times on Amicon^®^ Ultra 0.5 mL centrifugal filter units (MWCO 3 kDa, Merck, Darmstadt, Germany) to exchange SDS with PBS. The concentration of CH25H in the lysates was determined by the human C25H/CH25H ELISA Kit (LS-F7759, LSBio, Seattle, WA, USA) as per the manufacturer’s protocol. The sensitivity was 49 pg/mL, and the intra-assay and interassay precision was below 10% and 12%, respectively.

### 4.8. Western Blot

The protein content of FLS lysates was quantified using the Pierce™ BCA Protein Assay Kit (Thermo Fisher) according to the manufacturer’s protocol. Equal amounts of proteins were subjected to SDS-PAGE on an 8% Tris-Glycine gel for ABCA1 and 10% gels for the remaining proteins and blotted onto PVDF membranes in a Trans-Blot^®^ SD semi-dry electrophoretic transfer cell (Bio-Rad Laboratories, Munich, Germany) at 1.0 A/cm^2^ for 1.5 h.

Membranes were blocked with buffered 5% skimmed milk for 1 h at room temperature and then incubated with primary antibodies against CH25H (#NBP2-83971), sterol regulatory element-binding protein 1c (SREBP-1c, gene SREBF1, clone 2A4, RRID: AB_10001575) (both from Novus Biologicals, Wiesbaden, Germany), CYP7B1 (clone OTI1G7, #TA807549, OriGene, Herford, Germany), ABCG1 (clone ARC0336, RRID:AB_2849090, Thermo Fisher), HMGCR (clone CL0260, RRID:AB_2786973, Invitrogen, Karlsruhe, Germany), liver X receptor alpha (LXRα; gene NR1H3, clone PPZ0412, RRID:AB_2154888), ABCA1 (clone 1276B, #MAB72071), APOE (#AF4144), GAPDH (#2275-PC-100, RRID: AB_2107456), and ß-actin (clone 937215, #MAB8929) (the last 5 from R&D Systems, Wiesbaden, Germany).

Next, the membranes were incubated with polyclonal goat anti-rabbit, anti-mouse, or anti-goat HRP-conjugated secondary antibody (#HAF007, RRID: AB_357234, HAF008, RRID: AB_357235, and HAF017, RRID: AB_562588, respectively; R&D Systems). The immunoreactive bands on the blot were visualized on a chemiluminescence imager (ChemoCam Imager 3.2, Intas Instruments, Goettingen, Germany) using ECL Plus substrate (Amersham™, Cytiva, Freiburg, Germany). Some blots were stripped twice and reused to detect another protein, ß-actin, or GAPDH. Bands were quantified densitometrically in ImageJ (v1.53j; NIH) [42].

### 4.9. Statistical Analysis

Data are presented as means and standard deviations. For statistical analyses and graphics, we used R, version 4.0.3 [43], the R-package lattice [44], and other packages, as specified below. Each experimental condition was repeated 4–8 times using FLS from 5 to 9 patients. Each FLS culture per patient was propagated until Passage 4, aliquots of which were then randomly assigned to treatments.

For each of the 12 parameters (mRNA expression, PL release), the experimental setting was a randomized complete block design, with patient FLS culture as a block factor and a single treatment factor with 2–4 treatment levels. Normal q-q-plots, generated using the R-package car [45], did not reveal critical non-normality for the observed values.

The effects of treatments with only 2 levels were analyzed by Student’s paired *t*-test. In experiments with more than 2 treatment levels, the block factor was considered a random effect, and one-factorial mixed-effects ANOVA was performed using the R-package nlme [46]. Missing values, which were rare, were considered missing completely at random.

The analyses above were applied to ΔCt values for mRNA expression and % PL release to identify promising parameters in the studied family of 12 parameters. To adjust for multiple testing, the false discovery rate (FDR) was controlled by Benjamini’s and Yekutieli’s method [47] under dependency at 20%. Promising parameters identified by an FDR-adjusted *p*-value of less than 20% were analyzed further by post-hoc comparisons of their treatment levels with a stepdown version of Dunnett’s many-to-one comparisons or Westfall’s truncated closed test for Tukey’s all-pairwise comparisons, all of which are implemented in the R-package multcomp [48]. For promising parameters with only 2 treatment levels, the unadjusted *p*-values of Student’s paired *t*-test were used. Their significance threshold was set to 5%.

For the parameters that were analyzed by Western blot, the treatment effects were examined by Student’s paired *t*-test. Normal q-q-plots for absolute and relative differences did not reveal critical non-normality. The FDR in this family of paired *t*-tests was also controlled by Benjamini’s and Yekutieli’s method [47] at 20%.

## Data Availability

The data supporting the findings of this study are available from the corresponding author upon reasonable request.
